# Increase in the Tibial Slope Reduces Wear after Medial Unicompartmental Fixed-Bearing Arthroplasty of the Knee

**DOI:** 10.1155/2015/736826

**Published:** 2015-01-15

**Authors:** Patrick Weber, Christian Schröder, Jens Schwiesau, Sandra Utzschneider, Arnd Steinbrück, Matthias F. Pietschmann, Volkmar Jansson, Peter E. Müller

**Affiliations:** ^1^Department of Orthopedic Surgery, Physical Medicine and Rehabilitation, University Hospital, Ludwig Maximilians University (LMU), Campus Großhadern, Marchioninistraße 15, 81377 Munich, Germany; ^2^Aesculap AG Research & Development, Am Aesculap-Platz, 78532 Tuttlingen, Germany

## Abstract

*Introduction*. Unicompartmental arthroplasty of the knee in patients with isolated medial osteoarthritis gives good results, but survival is inferior to that of total knee prosthesis. Knees may fail because positioning of the prosthesis has been suboptimal. The aim of this study was to investigate the influence of the tibial slope on the rate of wear of a medial fixed-bearing unicompartmental knee arthroplasty. *Materials and Methods*. We simulated wear on a medial fixed-bearing unicompartmental knee prosthesis (Univation) in vitro with a customised, four-station, and servohydraulic knee wear simulator, which exactly reproduced the walking cycle (International Organisation for Standardisation (ISO) 14243-1:2002(E)). The medial prostheses were inserted with 3 different posterior tibial slopes: 0°, 4°, and 8° (*n* = 3 in each group). *Results*. The wear rate decreased significantly between 0° and 4° slope from 10.4 (SD 0.62) mg/million cycles to 3.22 (SD 1.71) mg/million cycles. Increasing the tibial slope to 8° did not significantly change the wear rate. *Discussion*. As an increase in the tibial slope reduced the wear rate in a fixed-bearing prosthesis, a higher tibial slope should be recommended. However, other factors that are influenced by the tibial slope (e.g., the tension of the ligament) must also be considered.

## 1. Introduction

Unicondylar arthroplasty of the knee (UKA) is a standard procedure nowadays for patients with osteoarthritis of the medial knee. The 20-year survival of this type of implant is reported to be up to 91% for the Oxford knee (Biomet, Bridgeton, GB) [1]. In comparison, the Swedish knee arthroplasty register reported that the revision rate of UKA is higher than that of total knee arthroplasty, and the number of UKA has diminished during recent years [2]. However, UKA has advantages over total knee arthroplasty (TKA) as it can be implanted using minimally-invasive techniques, rehabilitation is quicker, and the kinematics of the knee are closer to those of the physiological knee as all the ligaments (collateral and cruciate ligaments) are retained [3–6]. In TKA the anterior cruciate ligament is always resected and the posterior depending on the design. This is probably the reason why patients who have total arthroplasty in one knee and UKA in the other are more satisfied with the UKA [7, 8].

The reasons for revision of UKA were also analysed in the Swedish knee register and the data show that in more than half the reason was wear or loosening [2]. To reduce the amount of wear is therefore a major aim in improvement of the longevity of UKA. Positioning is a critical factor in the longevity of the implant, but various authors and the manufacturers recommend a tibial slope in a broad range up to 20° [9].

We have therefore analyzed the influence of the (posterior) tibial slope in UKA mobile-bearing design and shown that a tibial slope of 4° or 8° reduced the wear rate in a mobile-bearing UKA [10]. However, it was shown that in fixed-bearing total knee replacements the tibial slope had no influence on the degree of wear [11]. As far as kinematics are concerned, there is also a difference in mobile-bearing and fixed-bearing UKA [12]. The hypothesis of the study was therefore that increasing the tibial slope would decrease the wear rate in fixed-bearing UKA.

## 2. Materials and Methods

### 2.1. Prosthesis and Embedment

Fixed-bearing unicompartmental knee prostheses (Univation fixed, Aesculap AG, Tuttlingen, Germany) were used for the in vitro analysis of wear. The femoral components and the tibial trays were casted of cobalt-chromium (CoCr29Mo) alloy. The tibial liners were made of ultra-high molecular weight polyethylene (UHMWPE; GUR 1020; *β*-sterilised 25–40 kGy and packed under inert gas).

**Figure 1:**
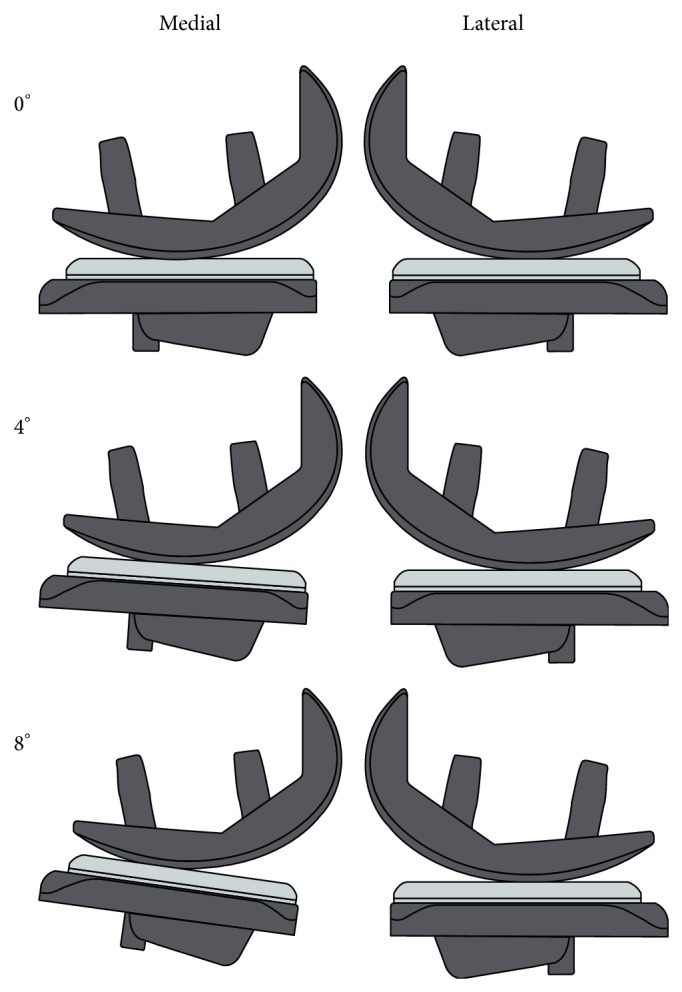
Alignment of the medial and lateral UKA in the knee wear simulator. Alignment of the posterior slope was changed by rotating the medial tibial tray. The lateral tibial tray and the position of the femoral components remained unchanged.

The medial tibial component was aligned at different slope angles (0°, 4°, and 8° posterior slope), while the lateral compartment remained with a constant slope of 0. The lateral component was implanted only to balance the varus/valgus positioning. The axis of rotation was the bisector of the maximum anterior-posterior expansion in the sagittal plane (Figure 1).

### 2.2. Wear Simulator Testing

For each position on the slope, three prostheses were tested with a customized servohydraulic knee wear simulator (EndoLab GmbH, Thansau, Germany). The wear simulator mimics walking in a plane for 5.0 million cycles as specified in the ISO-standard (ISO 14243-1:2002 Figure 2) recording anteroposterior translation (AP) and tibial internal-external rotation (IE) during the whole test period. In addition, one specimen was used as a load soak control by applying an axial load without flexion, AP force, or tibial torque. Lubrication between the components was guaranteed by a mixture of newborn calf serum with distilled water to reach a protein content of 30 g/L. Additives were used to stabilize the pH (EDTA, AppliChem, Darmstadt, Germany) and to avoid fungal growth (Amphotericin B Biochrom, Berlin, Germany) in the lubricant. Before the test was started all UHMWPE-liners were conditioned by storing the specimens in the lubricant until there was no further increase in weight.

Every 500,000 cycles the lubricant was replaced and the UHMWPE-liners were cleaned gently, and weighed using an analytical balance (Sartorius BP211D, Germany) with an accuracy of 0.01 mg according to the ISO 14243-2. Finally, the gravimetric wear was corrected with the load soak control and air buoyancy. After the wear tests, the UHMWPE-liners were examined with a reflected light microscope (Leica Microsystems, Heerbrugg, Switzerland) to qualify the wear pattern on the bearing surface.

### 2.3. Knee Kinematics

The kinematic data including the AP translation were analysed for a mean of the three test stations for ten full gait cycles at 5.0 million cycles. The cumulative AP translation was defined as the total of the AP movement between the femur and the tibia.

### 2.4. Particle Analysis

The lubricant was placed in a beaker and 37% hydrochloric acid was added at a ratio of 1 : 5. Digestion occurred by 60°C for 60 minutes. Afterwards, 1 mL of the digested solution was added to methanol 50 mL and vacuum filtered through a 20 nm aluminum oxide filter (Whatman, Anodisc, Maidstone, Great Britain). The filter was dried overnight in an exsiccator. Before we analyzed the surface of the filter by scanning electron microscopy (EVO LS 10, Carl Zeiss, Oberkochen, Germany), the filter was sputter-coated with a 3 nm layer of gold (SCD 050, Bal-Tec, Scotia, USA). Ten nonoverlapped pictures were taken at a magnification ×10,000 (29 nm/pixel) using a secondary electron detector. The shape and size of the wear debris found on the filter were then analyzed with imaging software (Qwin, Leica, Wetzlar, Germany) respecting ASTM F1877 standard and as previously reported [13].

### 2.5. Statistical Analysis

Statistical analysis was aided by IBM SPSS Statistics for Windows (version 22, Armonk NY, IBM Corp). All values are given as mean (SD). The rate of wear and the range of movement were tested for significance among the three groups (0°, 4°, and 8°). Before we chose a statistical test, the distribution was confirmed as normal (Kolmogorov-Smirnov-test) and analysis of variance (ANOVA) was used to assess the significance of differences among the groups. A Bonferroni post hoc test was then used to test the significance of differences between each single slope orientation (4° compared with 8°, 0° compared with 4°, and 0° compared with 8°). Probabilities of less than *P* < 0.05 were accepted as significant. The pattern of wear, characteristics of kinematics of the knee, and distributions of particles are presented descriptively.

## 3. Results

### 3.1. Rate and Pattern of Wear

By increasing the slope of the medial compartment the gravimetric rate of wear decreased significantly from 10.40 (SD 0.62) mg/million cycles at 0° slope to 3.22 (SD 1.71) mg/million cycles at 4° posterior slope (*P* < 0.01). A further increase in the tibial slope to 8° did not change the gravimetric wear rate on the medial side significantly (2.69 (SD 0.81) mg/million cycles; Figure 3; *P* = 1). When the slope in the medial compartment was changed the wear rate of the lateral liners remained unchanged (slope 0°: 5.13 (SD 0.91) mg/million cycles; slope 4°: 3.76 (SD 1.35) mg/million cycles; slope 8°: 4.13 (SD 0.41) mg/million cycles; *P* = 0.3).

Polishing characterized the wear pattern on all tibial liners across all tested angles of slope. We found only small scratches and no pitting or delamination on the worn surfaces.

### 3.2. Kinematics of the Knee

AP translation (*P* < 0.01) and IE rotation (*P* < 0.01) were both significantly reduced after the tibial slope had been increased to 4° or 8° (Figure 4). In the neutral position, the mean of the translation in the AP direction was 5.43 (SD 0.08) mm. This decreased to 2.83 (SD 0.70) mm at 4° tibial slope and increased slightly again to 3.80 (SD 0.07) mm for 8° tibial slope (*P* = 0.1). The curves of the AP movement (Figure 5(a)) were similar for all the tested slope alignments.

The IE rotation for 0° slope was 7.08° (SD 0.13°). It decreased to 2.91° (SD 0.13°) at 4° slope and rose again slightly to 4.15° (SD 0.45°) at 8° tibial slope (SD *P* < 0.01; Figure 4). The analysis of the curves of the IE rotation (Figure 5(b)) showed that the rotation from 0 to 4° was significantly decreased. This decrease remained for the 8° slope, but the difference was smaller.

### 3.3. Size and Shape of Particles

Particles were found with diameters (ECD) from 0.06 to 8 *µ*m for each angle of slope, while the frequencies of the distributions and the mean diameter were similar between the tested groups (Figure 6). There was no difference in the mean shape (aspect ratio : roundness) of the wear particles analyzed (Table 1).

## 4. Discussion

This study has shown that an increased tibial slope of 4° led to a reduction in the wear rate in fixed-bearing unicompartmental knee prosthesis in vitro. A further increase to 8° slope did not change the wear rate significantly. This was probably because the tibial rotation and the AP translation were reduced with a tibial slope of at least 4°. A further increase to 8° did not change the AP translation significantly.

The observed wear rate of 10.40 mg/million cycles at 0 slope is similar to that measured by Grupp et al. with the same prosthesis (10.54 mg/million cycles) [14]. Kretzer et al. also analyzed the wear of the Univation and showed a slightly lower wear rate of 7.51 mg/million cycles [15]. This difference may be because they used a different experiment with a different simulator and composition of the serum [16]. Laurent et al. found a comparable volumetric wear rate of 7.1 mm^3^/million cycles on the medial side under displacement control on an AMTI knee wear simulator [17]. The wear rate of the fixed-bearing knee was higher than the rate obtained by our study group with the same prosthesis in the mobile-bearing design with 0 slope (10.40 mg/million and 3.46 mg/million cycles, resp.) [10]. This is contrary to the results published by Kretzer et al, who found a reduced rate with the fixed-bearing prosthesis [15]. Interestingly, by increasing the tibial slope the difference between the wear rate for the fixed and mobile-bearing design lessened (8° tibial slope: 2.69 mg/million and 0.99 mg/million cycles, resp.) [10].

**Figure 2:**
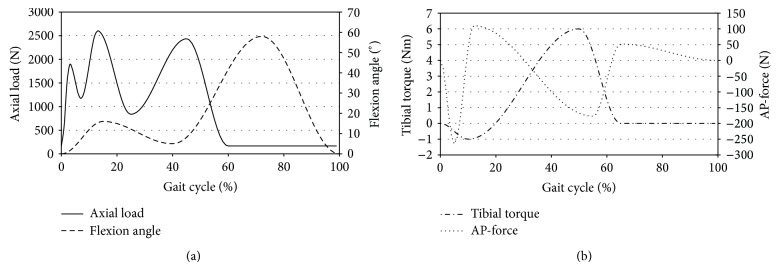
Applied axial load, flexion angle (both (a)), tibial torque, and AP-force (both (b)) during one cycle according to ISO 14243-1:2002.

**Figure 3:**
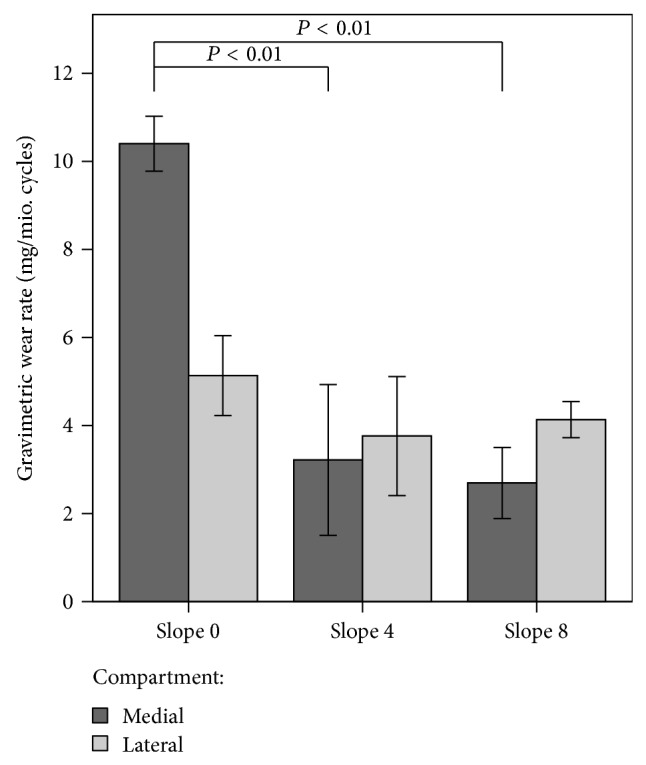
Wear rate of the tested specimens in different slope angles divided in a medial and lateral compartment. The differences between 0° slope and 4° slope and 0° slope and 8° slope were significant.

**Figure 4:**
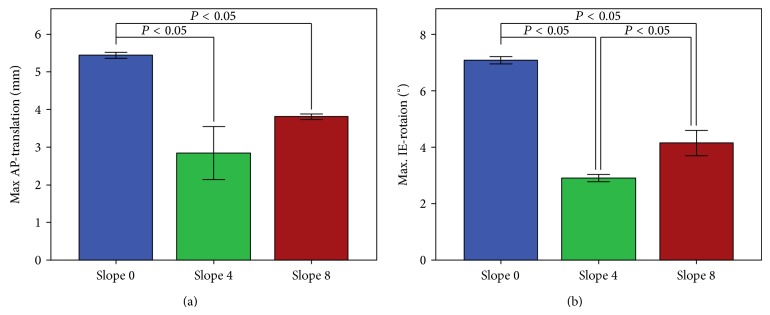
The maximum movement between the tibia and the femur for the different tibial slopes. The results are presented as means between 0 and 5 million cycles (every 500th cycle recorded); (a) = AP translation; (b) = IE rotation.

**Figure 5:**
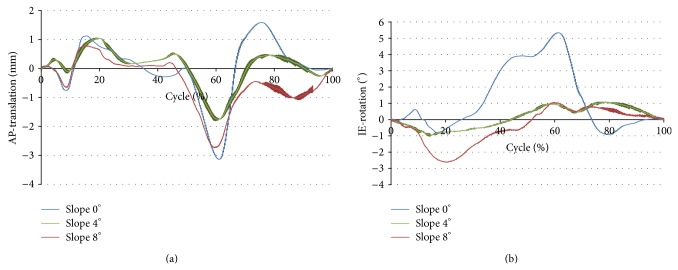
Knee kinematics out of 10 randomized cycles between 4.5 million and 5.0 million cycles of all 3 specimens. (a) AP translation: plus values define an anterior movement of the femur in relation to the tibia. There is slightly less movement between the femur and the tibia by increasing the tibia slope. (b) IE rotation: plus values define internal rotation of the tibia. There is significantly less rotation between the tibia and the femur at 4° and 8° tibial slopes. The thickness of the single plots shows ±SD.

**Figure 6:**
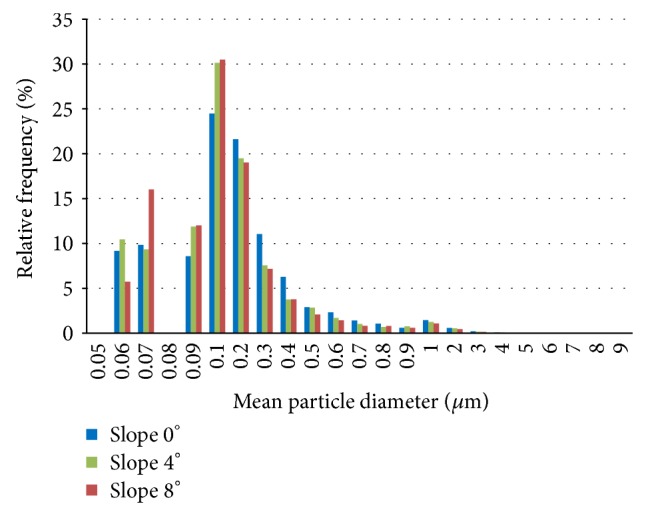
Frequency distribution of wear particles of the mean diameter for the different slope angles generated between 4.5 and 5.0 million cycles.

**Figure 7:**
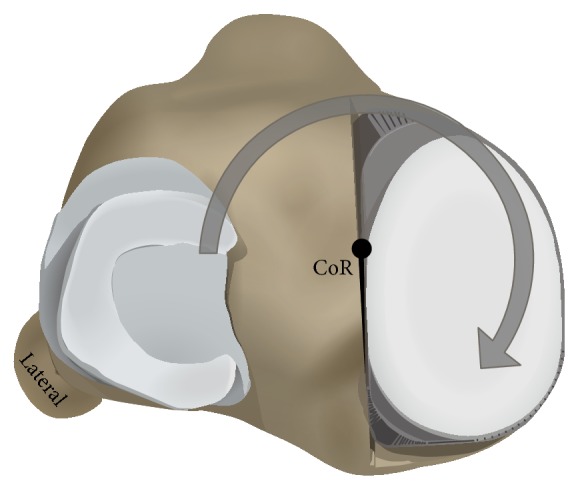
View from above of a left tibia with an implanted tibial component and the inlay on the medial side. Grey = the internal torque applied by the simulator. With an increased tibial slope in the medial compartment this force has to act against the tibial slope and the rotational movement is lessening. CoR = centre of rotation.

For the interpretation of the kinematic results it is important to consider the functioning of the knee simulator according to the ISO (human gait cycle). The internal torque is applied in the simulator between 20% and 60% of the gait cycle as during this moment of the gait cycle there is a physiological internal rotation of the tibia (Figures 2(b) and 7). This torque led to a rotation in the simulator with a neutral tibial slope (Figure 5(b), blue). By applying a tibial slope on the medial side, the internal rotation becomes less, as the torque force has to act against the tibial slope and is usually already neutralised with 4° of tibial slope (Figure 5(b) green and red, Figure 7). A higher tibial slope also led to less AP movement, but this difference was less pronounced between 0° and 4° slopes. A further increase to 8° slope again had a tendency towards increased AP translation compared with a 4° slope. This is probably a result of the complex movement in the knee joint (superimposition of different movements); a measurement error however cannot be excluded. This difference had no influence on the wear rate, particle size, or shape and wear pattern, so it is of little relevance.

The reduction of the movement between the tibia and the femur led to less wear, which is similar to that already seen in the mobile-bearing UKA [10]. The kinematics, however, differ between the fixed and mobile design. In the mobile-bearing UKA increasing tibial slope led particularly to a reduction of the AP movement [10], while in the fixed-bearing knee, this study showed that the reduction of rotation was more pronounced with the increased tibial slope. The reduction in tibial rotation is particularly interesting, as McEwen et al. showed a more parallel orientation of the macromolecules of ultra-high molecular weight polythene in the liner if multidirectional motions are prevented in a mobile-bearing TKA. They reported that this unidirectional movement led to less breaking out of the macromolecules from the polyethylene and with this to less wear [18]. This was probably also the case in the present study in the 4° and 8° tibial slope group.

The tibial slope in the human knee has a broad range with a mean of 9° [19, 20]. The testing with a higher tibial slope (4° and 8°, resp.) that led to a reduction in wear was closer to the physiological state as the neutral tibial slope. The observed reduced rotation of the knee with a higher tibial slope in the knee simulator is probably similar physiologically, where the tibial slope also leads to stabilisation of the knee.

A higher tibial slope can also be recommended to reduce the wear rate in the fixed-bearing UKA, but the tibial slope also influences many other factors in knee kinematics. It influences the ligamentous tension and the load on the proximal tibia and differs considerably among patients, and these must also be considered [19, 21–24]. In their retrospective analysis of UKA failures, Aleto et al. showed that in 15 out of 32 cases this was caused by collapse of the tibial component. Knees with anterior collapse showed a mean reduced tibial slope of 4.8°, while knees with dorsal collapse showed a higher tibial slope of 12.8°. The authors recommended a tibial slope of 7° [21]. In a finite element analysis, Sawatari et al. showed a reduced load in the cancellous bone stresses with a slope of 0 [23]. However, the study was a static computer model and the results were not confirmed experimentally. When they considered the influence on the ligaments in UKA, Hernigou and Deschamps showed that a higher tibial slope >13° lead to frequent ruptures of the anterior cruciate ligament [22].

A few limitations of the study need to be considered for the clinical interpretation of the findings. Firstly, wear simulation tests are time-consuming and therefore a limit of 3 specimens/group was tested. However, a pronounced reduction in wear rate together with altered knee kinematics was noted when a tibial slope of at least 4° was used, but this difference was not significant. Three samples were also used in similar studies of knee wear [13, 14, 25, 26].

Secondly, the UKA used is designed for the medial side. In the knee wear simulator it is necessary to test both condyles, so we therefore chose the same implant on the lateral side to create a placeholder during testing. This technique has also been established by other groups [14, 15, 25, 27].

Thirdly, the acting forces and moments in the knee may be changed with different alignments of the prosthesis. This possible difference was not quantified in this study. However, the input of the ISO-Standard was constant for all the groups. There are few other studies investigating malalignment in the simulator and here the ISO standard was used as well [11, 28].

Finally, the wear simulator mimicked only walking. It is known that other activities such as climbing stairs, squatting, and rising from a chair influence the wear [29]. However, these activities have not routinely been examined on knee wear simulators up to now, and most daily activity involves walking.

## 5. Conclusions

We have shown that compared with a 0 deg. tibial slope, a slope of 4 deg. led to reduced wear. A tibial slope of 8 deg. did not further reduce the wear rate. In the force-controlled knee wear simulator the higher tibial slope stabilized the fixed-bearing UKA and reduced the tibial rotation with an increasing tibial slope. Taking our evidence and other results from the literature into consideration and the influence of the tibial slope on other factors a tibial slope between 4 and 8° can be recommended.

## Figures and Tables

**Table 1:** Mean (SD) size and shape of the wear particles generated in different angles of slope.

	Mean diameter (*µ*m)	Equivalent circle diameter (*µ*m)	Aspect ratio	Roundness
Slope 0°	0.23 ± 0.27	0.18 ± 0.20	1.68 ± 0.74	0.58 ± 0.25
Slope 4°	0.20 ± 0.23	0.16 ± 0.16	1.61 ± 0.66	0.64 ± 0.26
Slope 8°	0.18 ± 0.21	0.15 ± 0.14	1.83 ± 0.76	0.53 ± 0.24
